# Severe Anaphylaxis From Remimazolam in a Mastocytosis Cancer Patient: A Case Report

**DOI:** 10.7759/cureus.69079

**Published:** 2024-09-10

**Authors:** Julie Mani, Amanda Strang, Adeel A Faruki, Zuhair A Siddiqui, Gabriel Mena

**Affiliations:** 1 Department of Anesthesiology and Perioperative Medicine, MD Anderson Cancer Center, Houston, USA; 2 Department of Anesthesiology and Perioperative Medicine, McGovern Medical School at UTHealth, Houston, USA

**Keywords:** anaphylaxis, anesthesiology, mastocytosis, oncology, remimazolam

## Abstract

Remimazolam besylate is a novel, rapid-onset, ultra-short-acting benzodiazepine with an advantageous cardio-respiratory safety profile. To date, there have been few published reports of remimazolam-induced anaphylaxis, none of which are documented in patients with mast cell disease. We describe the case of a 77-year-old male with oncologic mastocytosis who developed anaphylaxis following remimazolam administration. Symptoms included shortness of breath, hypoxia, loss of consciousness, tachycardia, and hypotension. Resolution of symptoms was attained after the administration of flumazenil, dexamethasone, diphenhydramine, famotidine, and phenylephrine. Suspicion of an anaphylactic reaction was confirmed by elevated serum tryptase levels. This case report helps establish how to diagnose anaphylaxis in a mastocytosis patient.

## Introduction

Remimazolam besylate is a novel, ultra-short-acting IV benzodiazepine gaining rapid popularity due to its rapid onset, short duration of action, and advantageous cardio-respiratory safety profile [1]. Remimazolam combines the gamma-aminobutyric acid (GABA) agonist properties of midazolam with the organ-independent metabolism of remifentanil [2]. The agent was initially approved in Japan in January 2020 and in the United States in July 2020. Since then, its use has been approved in China, Korea, Taiwan, the European Union, and the United Kingdom [1,3]. Given its rising popularity, it is imperative that anesthesiologists become informed of its utility and considerations for use. 

Like other benzodiazepines, remimazolam acts on gamma-aminobutyric acid-A (GABA-A) receptors to induce cell membrane hyperpolarization and provoke an influx of chloride ions. Neuronal activity decreases, thus making the drug a useful sedative, anterograde amnestic, and anticonvulsant [4]. Its structure is different, however, from common agents like midazolam due to the incorporation of a carboxylic ester side chain onto the benzodiazepine core. This structural modification allows non-specific tissue esterases to rapidly hydrolyze the compound into its physiologically inactive metabolite (CNS 7054) [1]. Remimazolam acts quickly, with an onset time of one to two minutes and recovery time of 10-40 minutes. In comparison, the onset of midazolam is typically three to five minutes, and recovery is 20-80 minutes [1]. These chemical properties together constitute the favorable anesthetic profile - "fast on, fast off" - quick sedation, short duration of action, and short recovery time.

Remimazolam offers several unique advantages for procedural sedation. Its safety has been clinically proven for procedural sedation in gastrointestinal endoscopy, bronchoscopy, and hysteroscopy in place of propofol [4]. In comparison to traditional sedatives, patients who receive remimazolam have higher procedural success rates, shorter recovery times, earlier discharge times, and decreased rates of bradycardia, hypotension, and respiratory depression [5]. Though well tolerated by most patients, the most common side effects include hemodynamic disturbances such as hypotension/hypertension and bradycardia/tachycardia. Other potential side effects include nausea, agitation, and confusion [6]. There are less than 15 documented case reports of remimazolam-induced anaphylaxis, none of which are documented in a patient with mast cell disease [7]. In this case report, we present the unique case of a patient with clinical mastocytosis who developed an anaphylactic reaction after remimazolam administration. 

## Case presentation

The patient was a 77-year-old male with a history of oncologic mastocytosis who presented to the interventional radiology suite for tunneled central line placement. The patient had an extensive past medical history which included renal failure, coronary artery disease, hypertension, stress-induced cardiomyopathy, left bundle branch block, and episodes of arrhythmias including supraventricular tachycardia and atrial fibrillation with rapid ventricular response. Notably, the patient reported that since his diagnosis of mastocytosis, he had experienced a few other allergic drug reactions including, but not limited to, Alka-Seltzer, acetaminophen, calcium carbonate, aspirin, morphine, and ibuprofen. Allergic symptoms ranged from nausea and vomiting to rash, hypotension, dyspnea, and anaphylaxis. 

The complete blood count (CBC) at the time of the event included WBC 5.4, RBC 3.27, and Hgb 12.4. Differential count was neutrophil 69%, lymphocyte 12%, monocyte 11.7%, eosinophil 1.9%, and basophil 0.6%. The coagulation panel of the patient was notable for a prothrombin time of 15.2 seconds, partial prothrombin time of 34.8 seconds, and INR level of 1.22. While prothrombin time and INR were slightly elevated, coagulation abnormalities are not uncommon in cancer patients. Medication history included Amiodarone (Pacerone) 200 mg PO daily, valacyclovir (Valtrex) 500 mg PO every other day, citalopram (Celexa) 20 mg PO daily, fluticasone propionate (Flonase) 50 mcg nasal spray daily, fexofenadine (Allegra) 30 mg PO daily, simvastatin (Zocor) 20 mg PO daily. As needed medications included tramadol (Ultram) 50 mg PO for pain and epinephrine (EpiPen) 0.3 mg/0.3 mL injection for anaphylaxis.

In the pre-procedure area, the patient was at his baseline health status with normal laboratory values and vital signs. Following transfer to the interventional procedure suite, standard anesthesiology monitors were applied, and sterile drapes were placed in preparation for central line placement. Upon sterile draping of the face, as is routinely applied for central line insertion, the patient became extremely anxious. The anesthesiologist responded by administering 2.5 mg of remimazolam while the interventional radiology proceduralist scanned the right internal jugular vein with a Sonosite ultrasound probe (FUJIFILM Sonosite, Bothell, Washington). One minute after administration of the remimazolam, the patient said "I can't catch my breath", and vital signs, which were initially stable with a blood pressure (BP) of 139/75 mmHg, heart rate (HR) of 86 bpm with baseline left bundle branch block on electrocardiogram, oxygen saturation (SpO2) of 98%, and respiratory rate (RR) of 13 breaths/minute, changed dramatically. The patient's respirations increased to 35 breaths/minute, heart rate increased to 103 bpm, and SpO2 decreased to 90%. The patient became unresponsive to commands, and cyanosis worsened, with a further drop in oxygen level to SpO2 of 87%. This clinical picture raised high suspicion for a perioperative anaphylactic reaction, with key features of perioperative anaphylaxis outlined below (Table 1).

**Table 1:** Detection of perioperative anaphylaxis *But must be differentiated from other causes of drop in end-tidal carbon dioxide

Detection of Perioperative Anaphylaxis	
Mechanisms of anaphylaxis	IgE mediated (60%)
	Non IgE mediated: IgG or complement activation
Non perioperative causes	Food, environmental triggers
Perioperative	Chlorhexidine, iodine
	Neuromuscular blockers
	Surgical dyes
	Chlorhexidine, iodine
Symptoms in awake patient	Cutaneous (itching, hives)
	Pulmonary (wheezing, dyspnea)
	Complaining of feeling of "doom"
Symptoms under anesthesia	With intubated and unconscious patient
	Cardiopulmonary symptoms
	· Hypotension, vasodilation
	· Tachycardia, later bradycardia in cases of severe hypoxia
	· Ventricular fibrillation
	Respiratory symptoms
	· Refractory bronchospasm and added breath sounds
	· Acute pulmonary edema
	· More severe correlated with end-tidal carbon dioxide (ETCO_2_)*

Bag-mask ventilation was initiated from our Datex Ohmeda anesthesia machine (Avante Health Solutions, Louisville, Kentucky). Two doses of flumazenil were administered: a 0.2 mg dose with a subsequent 0.1 mg dose. There was a further decline in the patient's blood pressure to 70/50 mmHg. The involvement of both the patient's respiratory and cardiovascular systems and the patient's nonresponse to flumazenil prompted us to start preparing our emergency intubation equipment as well as epinephrine to administer for anaphylactic shock. As that was prepared, the anesthesiologist quickly gave 10 mg of dexamethasone (Decadron), 25 mg of diphenhydramine (Benadryl), 20 mg of famotidine (Pepcid), and 100 μg of phenylephrine to the patient while starting Optiflow nasal high flow oxygen at 70 L/minute. Within minutes, the patient's vitals improved to a BP of 119/77 mmHg, HR of 115 bpm, and SpO2 of 94%. The patient began to follow commands, and the heart rate further normalized after the administration of 5 mg of esmolol.

The choice of giving phenylephrine before epinephrine, despite the latter being standard for anaphylaxis, was based on the patient's cardiac profile. Specifically, phenylephrine was deemed beneficial given the patient's low ejection fraction (ejection fraction of 25% noted on echocardiographic report), left bundle branch block, and propensity to tachycardic arrhythmias, such as his supraventricular tachycardia (SVT) history. Furthermore, the echocardiographic report noted both left and right ventricle dilation, left ventricular diastolic dysfunction, and right ventricular systolic dysfunction. During the preparation of epinephrine, we administered phenylephrine in the interim and noted a rapid improvement in the patient's symptoms. Once prepared, the epinephrine was continually available in case symptoms failed to resolve or returned, which did not happen in this case. 

A serum tryptase concentration was drawn and sent immediately following the incident. The results revealed a significantly elevated concentration of 251 ng/mL, far exceeding the normal range (which is considered negative when below 11.5 ng/mL). This confirmed the anaphylactic episode and response to treatment. Suffice it to say, when the patient returned for subsequent procedures in the interventional suite, he tolerated his anesthetic quite well sans remimazolam. A complicating red herring is that serum tryptases may be elevated in non-anaphylactic events in patients with mastocytosis; however, in general, a 15-20% rise from baseline tryptase elevation is considered positive. While the patient did not have a baseline tryptase level to compare to, verifying that there is a rise from baseline is a secondary confirmation for anaphylaxis in mastocytosis patients. Considering the patient's clinical symptoms, positive response to anaphylaxis protocol, and remarkably elevated tryptase level, we felt confident in the diagnostic value of the tryptase level as a marker of anaphylaxis in this patient (Table 2). Subsequent consultations with allergy and immunology experts nationwide have reinforced that even in mastocytosis patients, a twenty percent increase in tryptase from baseline would be considered confirmatory.

**Table 2:** Defining criteria for anaphylaxis confirmation in different patient populations

Establishing anaphylaxis diagnosis in routine vs mastocytosis patients	
Onset	Up to 20 minutes after drug administration
Positive diagnosis in normal patient	· Obtain serum tryptase level 30 minutes - 2 hours after start of reaction
	Greater than 9.8 µg/l highly sensitive
	Greater than 33 µg/l very specific
Positive diagnosis in mastocytosis	· obtain serum tryptase 15 to 60 minutes after episode resolution and 24+ hours after resolution to obtain baseline serum tryptase
	Suggested anaphylaxis: 1.2 x basal level + 2ng/mL

## Discussion

The incidence of perioperative anaphylaxis in the United States is 15.3 per 100,000 procedures [8], with a mortality rate between 1.4% to 6% [9]. Clinicians must identify symptoms within a reasonable time frame of antigen exposure to consider the reaction anaphylactic (Table 3). Severe hypotension alone is not a reliable indicator. Other clinical manifestations include urticaria, refractory bronchospasm, acute pulmonary edema, loss of consciousness, dysrhythmias, or even cardiac arrest [9]. While perioperative anaphylactic reactions to antibiotics (penicillin, cephalosporins, glycopeptides), neuromuscular blocking agents (succinylcholine, rocuronium), dyes (patent blue), and chlorhexidine [9] are well documented, emerging evidence suggests remimazolam be considered a potential allergen in rare cases. 

**Table 3:** Unique features of anaphylactic episodes in the mastocytosis patient

Anaphylaxis in the mastocytosis population	
Incidence all anaphylaxis episodes	4-6x greater than the general population
Incidence perioperative anaphylaxis	0.4% of adults with mastocytosis
2 phenotypes of anaphylaxis	Non-allergic histamine release
	IgE mediated hypersensitivity
Perioperative symptoms differ	· Cutaneous (erythema, rash, flushing)
	· Cardiovascular (vasodilation, hypotension)
	· Usually no bronchospasm
	· Gastrointestinal (nausea, vomiting)
Most common etiology	Non-Perioperative: Hymenoptera venoms (e.g., bee and wasp stings)
	Perioperative: no clear data on most common agents

Interestingly, patients allergic to remimazolam may demonstrate no allergy to midazolam. The synthetic colloid dextran 40 is unique in remimazolam; thus, patients with a rare dextran allergy may have a positive skin test to remimazolam but not midazolam [7,10,11]. While extremely uncommon, most cases of anaphylaxis to remimazolam occur during induction of general anesthesia when relatively larger doses (usually around 10 mg, one reports up to 97.5 mg) are administered [7]. In contrast, our patient experienced anaphylaxis at a 2.5 mg dose, raising the question of whether his mastocytosis increased his susceptibility to anaphylaxis.

Mastocytosis is a proliferative disorder of hematopoietic mast cell progenitors in the body, often in the skin, bone marrow, and gastrointestinal tract [12]. Mastocytosis occurs in one in 150,000 people, and symptoms are exacerbated by four categories of stress: psychological, pharmacological (i.e., atracurium, mivacurium, nefopam), mechanical (i.e., surgery, skin trauma), and thermal [13]. The mast cells release inflammatory mediators that induce dermatologic symptoms such as pruritus and erythema, cardiovascular symptoms such as tachycardia and hypotension, and gastrointestinal symptoms such as gastrointestinal reflux and vomiting [13]. Although the everyday risk of life-threatening reactions is low, reactions that compromise hemodynamic stability and airway patency deserve special consideration in a surgical setting.

Perioperatively, patients with mastocytosis rarely experience mast cell mediator-related symptoms (2%) or anaphylaxis (0.4%) [14]. Hypersensitivity signs typically manifest within minutes, presenting with cardiovascular and dermatological changes due to mediator release and mast cell burden on nearby tissue [13]. Debate surrounds prophylactic management; some recommend administering antimediator therapy, such as histamine antagonists and antileukotrienes, before every anesthetic procedure, while others recommend pre- and post-operative antimediator therapy to mitigate reaction risk [12]. Interestingly, given that psychological stress can induce a mastocytosis event, anti-anxiolytics are commonly administered to prevent a reaction [12]. Paradoxically, in our case, the anti-anxiolytic was the trigger for the anaphylactic event.

In case of a perioperative anaphylactic reaction, anesthesiologists should cease drug administration, call for assistance, and administer 100% oxygen. Epinephrine (10 to 50 μg) should be promptly given, along with crystalloid fluid bolus (20 to 30 ml/kg) for volume restoration. Beta2 agonist nebulizers can aid severe bronchospasm [9].

## Conclusions

This case report demonstrates a rare instance of anaphylaxis triggered by remimazolam, a sedative known for its low-risk profile and growing popularity. Recognition of early signs, including increased heart rate and respiratory rate and decreased blood pressure and oxygen saturation, enabled the timely application of anaphylactic protocol in the periprocedural suite. We describe the mechanism of hypersensitivity reactions in patients with oncologic mastocytosis and highlight key differences in offending agents, clinical presentations, and confirmatory tests with the general population. Anaphylactic reactions to remimazolam are rare. However, in the event of an anaphylactic reaction, anesthesiologists should include the drug in the differential diagnosis. 
